# Supplementary material for: Exposing Salmonella Senftenberg and Escherichia coli Strains Isolated from Poultry Farms to Formaldehyde and Lingonberry Extract at Low Concentrations
Source: Int J Mol Sci. 2023 Sep 26;24(19):14579. doi: 10.3390/ijms241914579 (PMC10572950; doi:10.3390/ijms241914579)
Supplement: Supplementary file 1 [file ijms-24-14579-s001.zip › ijms-2620404-supplementary.pdf]

Table S1. Survival of *S.* Senftenberg strains in biofilms after the treatment with formaldehyde at the concentrations of 0.02%, 0.2%, and 2.0% for 1 and 15 minutes

Strain No.	Formaldehyde concentration (%)	Formaldehyde action time (min)	CFU/mL	SD (±)	Bacterial survival (%)
131	0.0 (control)	0	5.48×10^9	4.63×10^7	100.0
	0.02	1	2.84×10^9	4.32×10^7	51.8
		15	2.57×10^9	4.71×10^7	46.8
	0.2	1	2.50×10^9	7.07×10^7	45.6
		15	1.82×10^9	8.22×10^7	33.2
	2.0	1	0	0	0
		15	0	0	0
132	0.0 (control)	0	3.27×10^9	9.70×10^7	100.0
	0.02	1	2.63×10^9	6.11×10^7	80.3
		15	2.65×10^9	6.24×10^7	80.9
	0.2	1	1.91×10^9	7.33×10^7	58.3
		15	1.43×10^9	4.71×10^7	43.8
	2.0	1	0	0	0
		15	0	0	0
133	0.0 (control)	0	2.80×10^9	8.07×10^7	100.0
	0.02	1	2.75×10^9	4.08×10^7	98.0
		15	1.84×10^9	9.60×10^7	66.0
	0.2	1	2.07×10^9	4.71×10^7	73.8
		15	1.48×10^9	7.17×10^7	52.8
	2.0	1	0	0	0
		15	0	0	0
134	0.0 (control)	0	2.75×10^9	9.09×10^7	100.0
	0.02	1	2.75×10^9	7.45×10^7	100.0
		15	2.39×10^9	7.35×10^7	87.0
	0.2	1	1.33×10^9	9.04×10^7	49.0
		15	5.35×10^8	5.00×10^6	19.0
	2.0	1	0	0	0
		15	0	0	0
135	0.0 (control)	0	2.57×10^9	6.76×10^7	100.0
	0.02	1	1.57×10^9	9.43×10^7	61.0
		15	1.50×10^9	8.16×10^7	58.4
	0.2	1	1.13×10^9	7.55×10^7	44.0
		15	5.00×10^8	7.07×10^6	19.5
	2.0	1	0	0	0
		15	0	0	0

Table S2. Survival of *E. coli* strains in biofilm after the treatment with formaldehyde at concentrations of 0.02%, 0.2%, and 2.0% for 1 and 15 minutes

Strain No	Formaldehyde concentration (%)	Formaldehyde action time (min)	CFU/mL	SD (±)	Bacterial survival (%)
WW01	0.0 (control)	0	6.80×10^9	6.73×10^7	100.0
	0.02	1	2.76×10^9	6.07×10^7	41.0
		15	2.80×10^9	2.91×10^7	41.0
	0.2	1	2.77×10^9	5.20×10^7	41.0
		15	1.92×10^9	9.69×10^7	28.0
	2.0	1	5.00×10^2	1.41×10^1	0.0
		15	0	0	0.0
WW02	0.0 (control)	0	3.16×10^9	7.87×10^7	100.0
	0.02	1	3.06×10^9	7.50×10^7	97.0
		15	2.37×10^9	5.00×10^7	75.0
	0.2	1	2.96×10^9	4.15×10^7	94.0
		15	1.93×10^9	9.42×10^7	61.0
	2.0	1	0	0	0.0
		15	0	0	0.0
KA01	0.0 (control)	0	3.86×10^9	4.31×10^7	100.0
	0.02	1	2.27×10^9	9.22×10^7	59.0
		15	2.11×10^9	6.63×10^7	55.0
	0.2	1	1.68×10^9	8.14×10^7	44.0
		15	1.48×10^9	9.27×10^7	38.0
	2.0	1	0	0	0.0
		15	0	0	0.0
CM02	0.0 (control)	0	3.31×10^9	8.19×10^7	100.0
	0.02	1	2.50×10^9	7.91×10^7	75.0
		15	2.38×10^9	8.29×10^7	72.0
	0.2	1	2.51×10^9	9.53×10^7	76.0
		15	2.39×10^9	6.63×10^7	72.0
	2.0	1	0	0	0.0
		15	0	0	0.0
CJ01	0.0 (control)	0	3.43×10^9	8.14×10^7	100.0
	0.02	1	2.65×10^9	5.72×10^7	77.0
		15	1.30×10^9	6.18×10^7	38.0
	0.2	1	1.52×10^9	7.23×10^7	44.0
		15	1.25×10^8	7.91×10^6	4.0
	2.0	1	1.10×10^3	7.07×10^1	0.0
		15	0	0	0.0
DP01	0.0 (control)	0	3.64×10^9	7.42×10^7	100.0
	0.02	1	3.47×10^9	6.98×10^7	95.0
		15	3.09×10^9	7.41×10^7	85.0
	0.2	1	2.58×10^9	5.12×10^7	71.0
		15	1.30×10^9	7.07×10^7	36.0
	2.0	1	0	0	0.0

	15	0	0	0.0
